# Perceiving Self, Others, and Events Through a Religious Lens: Mahayana Buddhists vs. Christians

**DOI:** 10.3389/fpsyg.2019.00217

**Published:** 2019-02-06

**Authors:** Tsung-Ren Huang, Yi-Hao Wang

**Affiliations:** Department of Psychology, National Taiwan University, Taipei, Taiwan

**Keywords:** religion, Christianity, Buddhism, attributional style, theory of mind, self–other

## Abstract

Are all religions essentially the same? Are believers of different religions heading in the same mental direction? To answer these questions from a sociopsychological perspective, we compared social sensitivity and causal attribution styles between Mahayana Buddhists, who practice unbiased love and compassion toward every being, and Christians, who pursue a union with God. Despite a similar cultural background, sex ratio, age distribution, socioeconomic status, and fluid intelligence level, these two religious groups in Taiwan showed opposite tendencies when inferring the mental states of others – as religiosity increased, the theory of mind ability increased in Mahayana Buddhists but decreased in Christians. Furthermore, these two religious groups showed opposite tendencies of attributional style – as religiosity increased, self-serving bias decreased in Buddhists but increased in Christians. These marked religiosity-dependent, sociopsychological effects suggest that different religions may shape or attract their followers who are moving in quite distinct mental directions.

## Introduction

In 2010, 83.6% of the 6.9 billion people around the world were religious, and by 2050, according to the Pew Research Center, the percentage will grow to 86.8% of a projected 9.3 billion people (Hackett et al., 2015). Religious beliefs and values thus play a considerable role in guiding the mind and behavior of the world population. Note that major religions share many elements in common, such as codes of ethics and the existence of supernatural being(s) who can be interacted with via specific rituals and exert power over life and the afterlife (Young, 2013). Thus, some researchers argue that different religions simply call the same deity by different names and their practices will eventually lead to the same spiritual destination (Hick, 1982).

However, different religions do teach fundamentally different worldviews and philosophies of life (Nicholson, 2016), which may affect how their adherents perceive themselves, others, and events. For example, in terms of the “self,” the no-self doctrine of Buddhism stands in sharp contrast with the concept of an unchanging, eternal soul in Christianity. In terms of “others,” Mahayana Buddhists practice universal compassion and love toward all sentient beings, while Christians cultivate an individual relationship with God. As another example, American Protestants tend to have more independent than interdependent selves and favor internal attributions more than Catholics do (Cohen, 2015).

Although different religions are often associated with different geographies and their corresponding cultures (Nisbett, 2004), religions can function as subcultures embedded within these broader cultural contexts to further shape the mind and behavior of religious adherents (Cohen, 2009). For example, while East Asians are more dialectical in reasoning and thus more tolerant of contradictions than are Westerners (Peng and Nisbett, 1999; Nisbett et al., 2001), it was found that subliminal Buddhist priming increased the tolerance of contradictions among Westerners with Christian backgrounds; however, subliminal Christian priming decreased the tolerance of contradictions among East Asians of Buddhist/Taoist backgrounds (Clobert et al., 2015). Overall, compared with Western-originated Christian religions, East Asian religions such as Buddhism emphasize low egocentrism and high interdependence/harmony, favor holistic thinking, and focus on similarities and consistencies among various kinds of relations (Clobert et al., 2014).

Do the abovementioned religious differences in values, beliefs, and attitudes actually moderate religious adherents’ capabilities of social cognition and social interaction? Theoretically, the ability to infer the mental states of others, namely, the Theory of Mind (ToM), is a prerequisite for successful human social interactions and can be achieved by putting oneself in another’s shoes (i.e., simulation theory) or by inferring from a mental theory, the causal rules of which are learned from past social interactions (i.e., theory theory). Therefore, a person’s ToM ability is likely modulated by how the person perceives oneself and/or interprets social events (i.e., attribution), both of which are taught quite differently in Mahayana Buddhism and Christianity. However, it remains to be empirically tested whether the followers of these two religions actually differ in their ToM abilities.

Past psychological studies of religions and the ToM mostly investigated the differences between religious and nonreligious groups rather than the differences among various religious groups. This body of literature provides converging evidence that religious beliefs are both the cause and the consequence of mind perception (Gervais, 2013) and often involve brain regions associated with ToM processing (Kapogiannis et al., 2009). For example, mentalizing ability is needed to personify God or gods (Norenzayan and Gervais, 2013), such as during personal prayers (Schjoedt et al., 2009), or to believe in a personal God in the first place (Norenzayan et al., 2012).

Note, however, that some of the results linking religions and the ToM should be interpreted with caution because of two methodological issues. First, the Reading the Mind in the Eyes Test (RMET) is a popular measure of the ToM ability but actually measures emotion recognition rather than the ToM ability (Oakley et al., 2016). Second, another popular measure of the ToM or cognitive empathy (Dvash and Shamay-Tsoory, 2014) is the Empathy Quotient (EQ) questionnaire (Lawrence et al., 2004; Wakabayashi et al., 2006), which can be subject to socially desirable responses and self-enhancement biases (e.g., Preti et al., 2011), particularly if responders are religious (Sedikides and Gebauer, 2010; Gebauer et al., 2017).

To revisit and extend previous research of religious effects on the ToM, we thus compared 200 Mahayana Buddhists to 200 Christians in Taiwan with regard to their mind-reading abilities using an accuracy-based measure of the ToM – the Yoni test (Shamay-Tsoory and Aharon-Peretz, 2007). Because the ToM ability may be closely related to self-centrality, we also measured these 400 research participants’ degrees of self-serving bias (SSB) using the Pragmatic Inference Task (Winters and Neale, 1985), which is less sensitive to response biases than is the popular Attributional Style Questionnaire (Seligman et al., 1979; Peterson et al., 1982).

Because Christians tend to be more self- and God-centered than other-centered (Han et al., 2008; Epley et al., 2009; Gebauer et al., 2017), we expect Christians to be more inclined toward self-serving attributions and less accurate in the ToM tasks than are Mahayana Buddhists. Moreover, we expect both the SSB and the ToM ability to be functions of religiosity. Specifically, the differences in self-other processing between Mahayana Buddhists and Christians, if any, are expected to grow with religiosity.

## Materials and Methods

### Procedure

Religious participants were recruited and introduced to the study website that presented all computerized questionnaires and assessment tests described in detail below. Informed consent was obtained online from all participants before enrollment in this study, which was approved by the Research Ethics Committee of the National Taiwan University.

### Participants

Two hundred Mahayana Buddhists were recruited from Tzu Chi (50 male, 50 female) and Fo Guang Shan (50 male, 50 female), the two largest Buddhist denominations in Taiwan. Another 200 Christians were recruited from the Presbyterian Church (50 male, 50 female) and The Church (50 male, 50 female), the two largest Christian denominations in Taiwan. All measurements in the study were obtained independently from these 400 samples. In the literature, a sample size larger than 50 participants per religious group was sufficient to show significant group differences in the ToM and Attributional Style (e.g., Lyon et al., 1994; Shamay-Tsoory and Aharon-Peretz, 2007).

A basic information questionnaire was used to assess the study participants’ demographics, including sex, age, monthly income, education level, occupation, and religion.

### Assessments of Religiosity

A Mandarin Chinese version of the Religious Commitment Inventory-10 (Worthington et al., 2003) was constructed by the authors using back translation. This inventory measures the degree to which an individual follows his or her religious values, beliefs, and practices and uses them in daily living.

The translated inventory was further abridged from 10 to 7 items to exclude one item (“My religious beliefs lie behind my whole approach to life.”) that was very similar to another item (“Religious beliefs influence all my dealings in life.”) and two items that were potentially affected by one’s socioeconomic status (“I make financial contributions to my religious organization.” and “I keep well informed about my local religious group and have some influence in its decisions.”). All items of this abridged questionnaire were internally consistent (Cronbach’s alpha = 0.89) and are listed in Supplementary Table S2.

A participant’s religiosity score was operationally defined as the total score of all seven items, each of which was rated using a five-point Likert scale from 1 (“Strongly disagree”) to 5 (“Strongly agree”).

### Assessments of Fluid Intelligence

A computerized, 24-item version of the Raven’s Standard Progressive Matrices (RSPM) test (Bilker et al., 2012) was used to estimate fluid intelligence. Similar to Jigsaw puzzles, each test item asked a participant to identify one out of six or eight local pieces that could consistently complete a global pattern, which was presented in the form of either a 2 × 2 or a 3 × 3 matrix with one missing element.

A participant’s fluid intelligence score was operationally defined as the number of correct items.

### Assessment of Theory of Mind

A Mandarin Chinese version of the Yoni test (Supplementary Figure S1) was constructed by the authors using back translation. This sociocognitive test assessed participants’ ability to infer the mental states of a cartoon character named “Yoni.” The Yoni test has the advantage of assessing both cognitive and affective ToM (Kalbe et al., 2010) and has been used as an alternative measure to the other popular ToM tests in past studies (e.g., Bodden et al., 2010; Kidd and Castano, 2013).

Each trial of the test presented a particular combination of eye gaze and facial expression in Yoni’s face in the middle of the screen, together with four stimuli in the corners of the screen. In the first-order mentalization condition, these four stimuli were exemplars of a single semantic category (e.g., fruit or animal). In the second-order mentalization condition, each of the four exemplars in the screen corners was accompanied by a Yoni-like face with a particular combination of eye gaze and facial expression targeting its neighboring exemplar. In each trial, a study participant was asked to best describe Yoni’s mental state by choosing one of the four stimuli to complete a sentence presented at the top of the computer screen, such as “Yoni is thinking of _____.” in the first-order cognitive mentalization condition, and “Yoni loves the toy that _____ loves.” in the second-order affective condition.

We followed the standard procedure of the Yoni test that consisted of 66 trials and was divided into 8 first-order cognitive, 24 second-order cognitive, 8 first-order affective, 12 second-order affective, 8 first-order physical, and 6 second-order physical conditions, where the physical trials served as a control condition that required judgments of physical rather than mental states, such as “Yoni is close to _____.” in the first-order physical condition, and “Yoni has the chair that _____ has.” in the second-order physical condition. A participant’s ToM score for each type was obtained as the number of correct trials in each condition with all the physical trials excluded. The total ToM score was defined as the number of correct ToM trials, ranging from 0 to 52.

### Assessment of Self-Serving Attributional Styles

A Chinese version of the computerized Pragmatic Inference Task (Supplementary Box S1) was constructed by the authors using back translation. It was used to assess participants’ implicit attributional style. It was composed of six positive scenarios and six negative scenarios, each followed by four questions. Among the four questions, one was a causal attribution question reflecting either on external or internal locus of causality, and the other three questions required either factual or noncausal inferences. Four scores of attributional styles were calculated simply by counting the number of internal-locus or external-locus responses to positive or negative scenarios. Each score ranged from 0 to 6.

The tendency of causal attribution in favor of oneself could be quantified by the SSB that were computed by subtracting internal-locus scores of negative events from internal-locus scores of positive events (Lyon et al., 1994), resulting in values ranging from -6 to 6.

## Results

### Group Composition

The Buddhist and Christian groups did not differ significantly in any of our control variables. For example, they did not differ significantly in the distributions of sex, age, monthly income, education level, and occupation (Supplementary Table S1). Moreover, various forms of measurement invariance were confirmed (Supplementary Table S3) to ensure that our measured religiosity could be compared across the two religious groups (Milfont and Fischer, 2010); we found that the religiosity scores of the Buddhist group (*M* = 24.75, *SD* = 4.86) and the Christian group (*M* = 24.96, *SD* = 4.91) did not differ significantly (two-tailed two-sample *t*-test, *t* = 0.44, *p* = 0.66). The fluid intelligence scores of the Buddhist group (*M* = 18.97, *SD* = 2.66) and the Christian group (*M* = 19.19, *SD* = 2.73) did not differ significantly (two-tailed two-sample *t*-test, *t* = 0.82, *p* = 0.42) either.

### Perceiving Others

Using the Yoni test to assess the ToM abilities of our study participants, we found that Mahayana Buddhists and Christians differed significantly in their ToM performance, as shown in Table 1. Specifically, Mahayana Buddhists outperformed Christians in all aspects of the ToM, including the first-order ToM (i.e., inferring another person’s thoughts), the second-order ToM (i.e., inferring what one person thinks about another person’s thoughts), the cognitive ToM (i.e., inferring the cognitive mental states of others, such as intentions), and the affective ToM (i.e., inferring the affective mental states of others, such as emotions).

**Table 1:** Participants’ scores on the Yoni test.

	Mahayana Buddhists (*N* = 200)		Christians (*N* = 200)		*t*	*p*
	
	*M*	*SD*	*M*	*SD*		
			
First-order ToM	13.34	1.66	10.99	2.43	11.27	<0.001
Cognitive	6.74	1.19	5.60	1.25	9.34	<0.001
Affective	6.60	1.24	5.39	1.80	7.78	<0.001
Second-order ToM	30.70	2.53	28.39	2.76	8.72	<0.001
Cognitive	20.34	2.20	19.67	2.12	3.08	0.002
Affective	10.36	1.10	8.72	1.20	14.27	<0.001
Total cognitive ToM	27.08	2.95	25.27	2.77	6.30	<0.001
Total affective ToM	16.96	1.91	14.11	2.24	13.67	<0.001
Total ToM	44.03	3.79	39.38	4.49	11.21	<0.001


*Overall, Mahayana Buddhists earned higher scores than did Christians in all types of ToM tasks (one-tailed two-sample t-tests). See the text for more details*.

**FIGURE 1:**
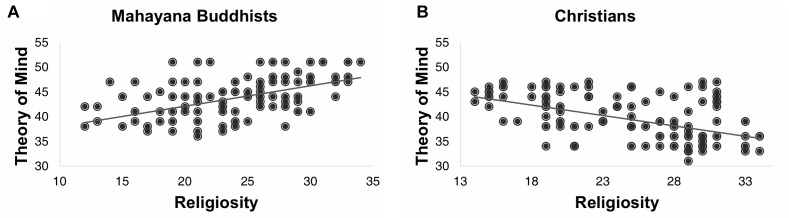
Theory-of-mind scores of **(A)** 200 Mahayana Buddhists and **(B)** 200 Christians as a function of religiosity scores. In both figure panels, each dot represents one individual, and each line results from a simple linear regression of the corresponding dots.

The observed differences in the ToM scores between Mahayana Buddhists and Christians could be explained in part by religious differences but not by fluid intelligence. Consistent with previous studies, we found that the ToM scores, regardless of religion, were positively correlated with the RSPM scores (Supplementary Table S4). However, the ToM scores correlated with the religiosity scores positively for Mahayana Buddhists (Pearson’s correlation *r* = 0.53, *p* < 0.001) but negatively for Christians (Pearson’s correlation *r* = -0.46, *p* < 0.001), as shown in Figure 1 and Supplementary Table S5. These ToM–religiosity correlations remained highly significant in multiple linear regressions (Supplementary Tables S6, S7) that controlled for sex, age, and fluid intelligence, which are factors known to affect the ToM (Henry et al., 2013; Ibanez et al., 2013); these results were partially replicated with a U.S. population on Amazon Mechanical Turk (Supplementary Table S11).

### Perceiving Events and Self

In addition to the ToM, Mahayana Buddhists and Christians also differed significantly in their attributional styles. Compared to Mahayana Buddhists, overall, Christians tended to explain events in favor of themselves by preferentially attributing positive events to internal/personal causes and preferentially attributing negative events to external/situational causes, as shown in Table 2. In other words, such a commonly seen SSB was relatively stronger in Christians than in Mahayana Buddhists.

**Table 2:** Participants’ scores on the Pragmatic Inference Task.

	Mahayana Buddhists (*N* = 200)		Christians (*N* = 200)		*t*	*p*
	
	*M*	*SD*	*M*	*SD*		
			
Positive-internality	2.95	1.73	4.37	1.41	-8.97	<0.001
Positive-externality	3.05	1.73	1.64	1.41	8.97	<0.001
Negative-internality	3.99	1.44	2.88	1.64	7.24	<0.001
Negative-externality	2.01	1.44	3.13	1.64	-7.24	<0.001
Self-serving bias	-1.04	2.46	1.49	2.45	-10.32	<0.001


*Overall, Mahayana Buddhists exhibited less SSB than did Christians (two-tailed two-samplet-tests)*.

**FIGURE 2:**
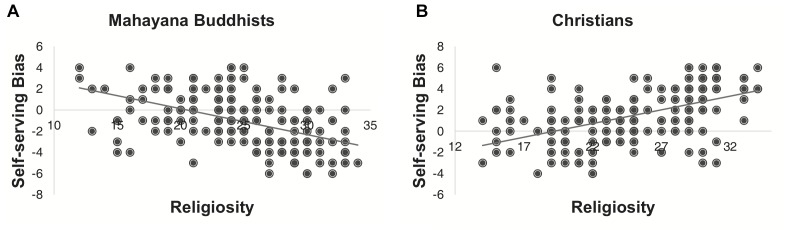
Self-serving-bias scores of **(A)** 200 Mahayana Buddhists and **(B)** 200 Christians as a function of religiosity scores. In both figure panels, each dot represents one individual, and each line results from a simple linear regression of the corresponding dots.

The observed differences in the SSB scores between Mahayana Buddhists and Christians could also be explained in part by religious differences. As shown in Figure 2 and Supplementary Table S8, the SSB scores were negatively correlated with religiosity scores for Mahayana Buddhists (Pearson’s correlation *r* = -0.49, *p* < 0.001) but positively correlated with religiosity scores for Christians (Pearson’s correlation *r* = 0.52, *p* < 0.001). These SSB–religiosity correlations remained highly significant in multiple linear regressions (Supplementary Tables S9, S10) that controlled for sex, age, and fluid intelligence, which potentially affected the SSB scores (Mezulis et al., 2004).

### Self–Other Processing

**FIGURE 3:**
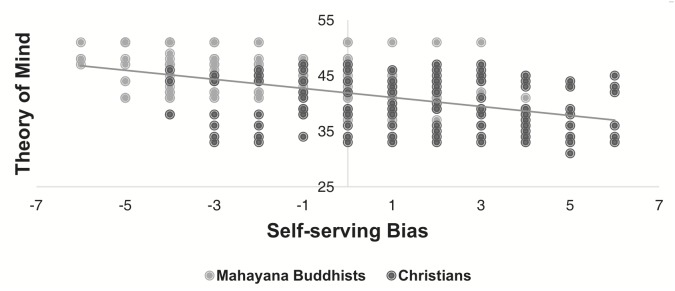
The ToM score as a function of the SSB score (*N* = 400 participants). Each dot represents one individual, and the line results from a simple linear regression of these dots.

Overall, the SSB scores were negatively correlated with the ToM scores regardless of religion (Pearson’s correlation *r* = -0.47, *p* < 0.001), as shown in Figure 3. In other words, more egocentric participants tended to be worse at understanding others.

## Discussion

In summary, compared to Christians, Mahayana Buddhists exhibited less SSB in causal attribution and better ToM capabilities. These sociopsychological differences between the two religious groups increased as a function of religiosity, suggesting religious differences to be a key contributor to the effects. It is possible that the practice of no-self and universal compassion/love directs Mahayana Buddhists’ thinking more outwardly toward others, whereas the focus on the soul and a close relationship with God directs Christians’ thinking more inwardly toward the self.

The negative correlation between Christians’ ToM scores and religiosity levels in our study appears to contradict with the previously observed positive relationship between the mentalizing ability and belief in God (e.g., Norenzayan et al., 2012; Willard and Norenzayan, 2013). Despite Christianity being the predominant religion in North America but a minor religion in Taiwan, the discrepancy in results was not primarily due to such a majority–minority or other cultural differences because we replicated our finding with a general U.S. population on Amazon Mechanical Turk.

Such a discrepancy may arise from methodological differences. Because we employed the Yoni test instead of the popular RMET and the EQ questionnaire, it is likely that different measures of mentalizing ability assess different components of cognitive processes (Bodden et al., 2010; Norenzayan et al., 2012; Kidd and Castano, 2013; Oakley et al., 2016). For example, the image-based RMET employed in previous studies requires a *perceptual* understanding of facial expressions, whereas the cartoon-based Yoni test employed in our study requires a *cognitive* understanding of situations. Additionally, because more religious Christians are more prone to self-enhancement biases (Sedikides and Gebauer, 2010; Gebauer et al., 2017), they might overstate their mentalizing abilities when answering to questions such as “I really enjoy caring for other people,” “I find it easy to put myself in somebody else’s shoes,” and “I am good at predicting what someone will do” in the EQ questionnaire, thus showing a positive relationship between mentalizing and religious belief.

As to attributional styles, our finding – more religious Christians are more likely to make self-serving attributions – is consistent with the self-centrality tendency observed in the previous literature (e.g., Epley et al., 2009; Gebauer et al., 2017), and its counterpart – more religious Mahayana Buddhists are less likely to make self-serving attributions – adds to the literature by showing that such a self-centrality tendency is not shared across religions. Albert Einstein once said that “*Scientific research is based on the idea that everything that takes place is determined by laws of Nature, and therefore, this holds for the action of people. For this reason, a research scientist will hardly be inclined to believe that events could be influenced by a prayer, i.e. by a wish addressed to a Supernatural Being*” (Einstein, 2013). Although a prayer *per se* may not influence an event, we have shown that individuals who pray may perceive the unfolding of the same event differently because of their religion-associated attributional styles. After all, the actions of individuals are determined not only by laws of Nature but also by laws of psychology.

Overall, our correlational findings – the opposite tendencies of self-other processing between Mahayana Buddhists and Christians in Taiwan – can be interpreted from different causal directions. While religions as meaning systems may shape the mind and behavior of their followers (Silberman, 2005), these followers may as well be attracted to different religions because of their predispositions (Saroglou, 2012) or cultural backgrounds (Cohen, 2009). As a result, religious affiliation or religiosity, despite being treated as explanatory variables in the present study, are themselves outcome variables of multiple factors.

In either causal direction, we can confirm that Taiwanese Mahayana Buddhists and Christians exhibit distinct sociopsychological differences in self–other processing that increase as a function of religiosity. These results suggest that different religions are superficially similar but can be quite distinct at their core. As a consequence, the more religious their adherents are, the more divergent mental destinations they may move toward.

## Author Contributions

T-RH and Y-HW contributed equally to this research. Both authors conceived the study. Y-HW collected the data under the supervision of T-RH. Both authors analyzed the data, interpreted the results, and wrote the manuscript.

## Conflict of Interest Statement

The authors declare that the research was conducted in the absence of any commercial or financial relationships that could be construed as a potential conflict of interest.
