# Supplementary material for: Perceiving Self, Others, and Events Through a Religious Lens: Mahayana Buddhists vs. Christians
Source: Front Psychol. 2019 Feb 6;10:217. doi: 10.3389/fpsyg.2019.00217 (PMC6373579; doi:10.3389/fpsyg.2019.00217)
Supplement: Supplementary file 1 [file Table_1.DOCX]

**Type:** Supplementary Materials

**Title:** Perceiving Self, Others, and Events through a Religious Lens: Mahayana Buddhists vs. Christians

**Short Title:** Perceiving Self, Others, and Events via Religion

**Author Affiliation:**

Tsung-Ren Huang ([tren@mil.psy.ntu.edu.tw](mailto:tren@mil.psy.ntu.edu.tw))

Yi-Hao Wang ([yhwang@mil.psy.ntu.edu.tw](mailto:yhwang@mil.psy.ntu.edu.tw))

Department of Psychology, National Taiwan University

No. 1, Sec. 4, Roosevelt Rd., Taipei 10617, Taiwan

**Corresponding Author:**

Tsung-Ren Huang ([tren@mil.psy.ntu.edu.tw](mailto:tren@mil.psy.ntu.edu.tw))

Address: No. 1, Sec. 4, Roosevelt Rd., Taipei 10617, Taiwan

Phone: +886-2-3366-3104

**Keywords:** Religion, Christianity, Buddhism, Attributional Style, Theory of Mind, Self-Other

**Supplementary Materials**

***Participants***

|  | Mahayana Buddhists  (*N* = 200) | | Christians  (*N* = 200) | | *t* */χ^2^* | *p* |
| --- | --- | --- | --- | --- | --- | --- |
|  | *M* | *SD* | *M* | *SD* |  |  |
| Age (years) | 45.46 | 10.84 | 44.72 | 11.61 | 0.66 | .51 |
| Income |  |  |  |  | 11.44 | .25 |
| Education |  |  |  |  | 7.03 | .14 |
| Occupation |  |  |  |  | 9.73 | .37 |

**Table S1.** Demographics of the study participants and comparisons between the Buddhist and Christian groups using a two-tailed two-sample *t*-test for Age and Chi-squared tests for categorical distributions of Income, Education, and Occupation.

***Assessments of Religiosity***

| Item | Unique-nesses | Item-total  correlation |
| --- | --- | --- |
| 1. I often read books and magazines about my faith. | .39 | .81 |
| 2. I spend time trying to grow in understanding of my faith. | .54 | .75 |
| 3. Religion is especially important to me because it answers  many questions about the meaning of life. | .42 | .79 |
| 4. I enjoy spending time with others of my religious affiliation. | .63 | .69 |
| 5. Religious beliefs influence all my dealings in life. | .36 | .81 |
| 6. It is important to me to spend periods of time in private  religious thought and reflection. | .27 | .85 |
| 7. I enjoy working in the activities of my religious affiliation. | .62 | .70 |

**Table S2**. All items used to measure religiosity (N=400 participants). Uniquenesses calculated the proportion of an item's response variance that was not shared with the responses of other items in a factor analysis with religiosity being the only factor.

| *Type of factor constraints shared across the two groups* | *DF* | *χ^2^* | *χ^2^*  *DIFF* | *D*  *DIFF* | *p* |
| --- | --- | --- | --- | --- | --- |
| 1. structure | 28 | 66.12 |  |  |  |
| 2. structure+loadings | 34 | 76.58 | 10.46 | 6 | .11 |
| 3. structure+loadings+intercepts | 40 | 81.38 | 4.80 | 6 | .57 |
| 4. structure+loadings+intercepts+residual vairances | 47 | 90.95 | 9.57 | 7 | .21 |
| 5. structure+loading+intercepts+residual variances+means | 48 | 91.27 | 0.31 | 1 | .58 |

**Table S3**. Chi-square difference tests for factor equivalence across the two religious groups (N=400 participants). Five sequentially tested models were reported and each more restricted model was compared with the previous one by a chi-square difference test. These restricted models did not lead to significant worse fits than the baseline model (i.e., the one assuming an equal factor structure). DF=degrees of freedom; DIFF= difference.

***Assessment of Theory of Mind***

|  | 1^st^-order | 2^nd^-order |
| --- | --- | --- |
| Cognitive | 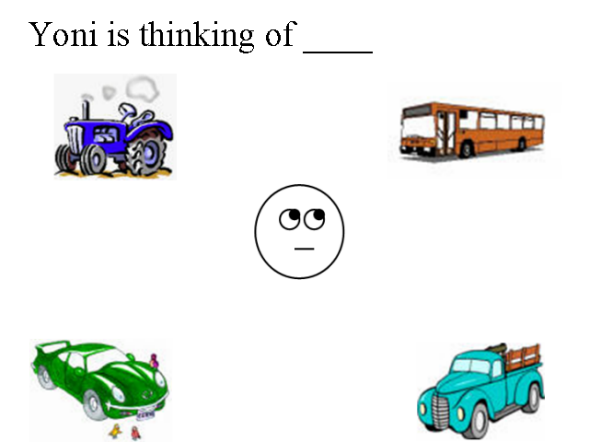 | 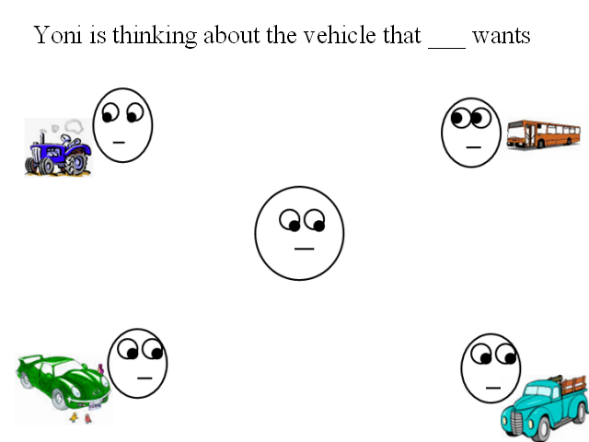 |
| Affective | 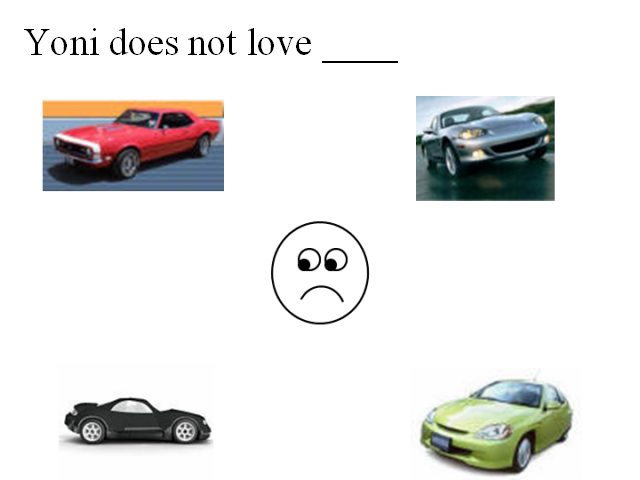 | 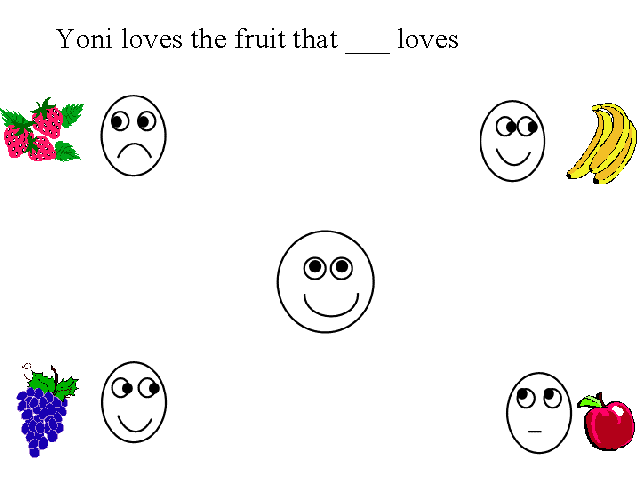 |
| Physical | 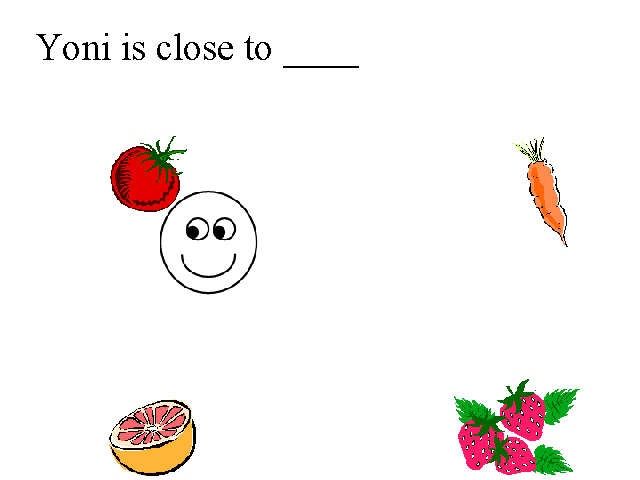 | 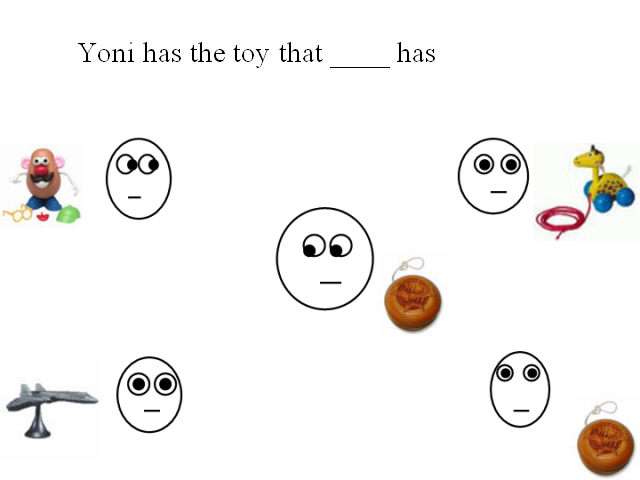 |

**Fig. S1**. Example trials from the Yoni test (Shamay-Tsoory & Aharon-Peretz, 2007).

|  | *Correlation with Fluid Intelligence* | | | |
| --- | --- | --- | --- | --- |
|  | Mahayana Buddhists  (N=200) | | Christians  (N=200) | |
| 1^st^-order metallization | | .37^***^ | | .19^**^ |
| Cognitive ToM | | .40^***^ | | .09 |
| Affective ToM | | .11 | | .20^**^ |
| 2^nd^-order metallization | | .24^***^ | | .45^***^ |
| Cognitive ToM | | .29^***^ | | .41^***^ |
| Affective ToM | | -.04 | | .31^***^ |
| Total Cognitive ToM | | .38^***^ | | .35^***^ |
| Total Affective ToM | | .04 | | .33^***^ |
| Total ToM | | .32^***^ | | .38^***^ |

*Notes:* **p* < .05, ***p* < .01, ***p < .001

**Table S4.** Pearson’s correlation between the ToM and RSPM scores*.*

|  | *Correlation with Religiosity* | | | |
| --- | --- | --- | --- | --- |
|  | Mahayana Buddhists  (N=200) | | Christians  (N=200) | |
| 1^st^-order metallization | | .42^***^ | | -.22^**^ |
| Cognitive ToM | | .22^**^ | | -.20^**^ |
| Affective ToM | | .34^***^ | | -.16^*^ |
| 2^nd^-order metallization | | .52^***^ | | -.55^***^ |
| Cognitive ToM | | .47^***^ | | -.64^***^ |
| Affective ToM | | .26^***^ | | -.13 |
| Total Cognitive | | .44^***^ | | -.58^***^ |
| Total Affective ToM | | .37^***^ | | -.20^**^ |
| Total ToM | | .53^***^ | | -.46^***^ |

*Notes:* **p* < .05, ***p* < .01, ***p < .001

**Table S5**. Pearson’s correlation between the ToM and religiosity scores*.*

The perceptual performance was defined as the scores of the "physical" condition in the Yoni test that asked experiment participants to infer the physical rather than mental states of Yoni.

| *Regressor* | *B* | *SE B* | *β* | *t* | *p* |
| --- | --- | --- | --- | --- | --- |
| Intercept | 27.59 | 2.90 | .07 | 0.86 | .39 |
| Sex | -0.54 | 0.45 | -.14 | -1.21 | .23 |
| Age | -0.02 | 0.02 | -.06 | -1.05 | .30 |
| Religiosity | 0.37 | 0.05 | .48 | 7.82 | <.001^***^ |
| Intelligence | 0.29 | 0.09 | .20 | 3.37 | <.001^***^ |
| Perception | 0.27 | 0.19 | .08 | 1.40 | .16 |

*Notes:* Final Model: *F* (5, 194) = 19.30, *p* < .001, adjusted *R^2^* = .32. Coding: 0=Male, 1=Female. **p* < .05, ***p* < .01, ***p < .001

**Table S6.** Regression model that predicted the ToM scores of Mahayana Buddhists (N=200).

| *Regressor* | *B* | *SE B* | *β* | *t* | *p* |
| --- | --- | --- | --- | --- | --- |
| Intercept | 36.76 | 3.97 | -.06 | -0.67 | .50 |
| Sex | 0.51 | 0.55 | .11 | 0.94 | .35 |
| Age | 0.02 | 0.02 | .05 | 0.77 | .44 |
| Religiosity | -0.35 | 0.06 | -.38 | -6.02 | <.001^***^ |
| Intelligence | 0.46 | 0.10 | .28 | 4.37 | <.001^***^ |
| Perception | 0.12 | 0.21 | .04 | 0.57 | .57 |

*Notes:* Final Model: *F* (5, 194) = 15.75, *p* < .001, adjusted *R^2^* = .27. Coding: 0=Male, 1=Female. **p* < .05, ***p* < .01, ***p < .001

**Table S7.** Regression model that predicted the ToM scores of Christians (N=200).

***Assessment of*** ***Self-serving Attributional Styles***

**A.** You decide to open your own dry cleaning shop in a small but growing town near the border. Your store will be the only one of its kind for miles around. In the first year of business, the town's population doubles and your business prospers. Your ad campaign is a big success and reactions from your customers indicate that the cleaning is quality work. Your gross sales exceed expectations. You wonder whether it would be to your advantage to open a chain of stores, so you go to the bank and apply for a loan. As you hoped, the bank approves the loan.

**A1.** What kind of store do you open?

A. Hardware

B. Dry cleaning

**A2.** In what part of the country is the town located?

A. Midwest

B. South

**A3.** Where is the loan obtained?

A. Loan agency

B. Bank

***A4.** What is the reason for the success of your business?

A. You are a smart businessman

B. You had no competition

**Box S1.** An example scenario from the Pragmatic Inference Task (Winters & Neale, 1985). The starred question is the critical question, which assesses a responder’s style of causal attribution.

|  | *Correlation with Religiosity* | |
| --- | --- | --- |
|  | Mahayana Buddhists  (N=200) | Christians  (N=200) |
| Positive-Internality | -.39^***^ | .40^***^ |
| Positive-Externality | .39^***^ | -.40^***^ |
| Negative-Internality | .36^***^ | -.43^***^ |
| Negative-Externality | -.36^***^ | .43^***^ |
| Self-serving Bias | -.49^***^ | .52^***^ |

*Notes:* **p* < .05, ***p* < .01, ***p < .001

**Table S8**. Pearson’s correlation between the scores of attributional styles and religiosity*.*

| Regressor | *B* | *SE B* | *β* | *t* | *P* |
| --- | --- | --- | --- | --- | --- |
| Intercept | 6.15 | 1.34 | -0.16 | -1.87 | .06 |
| Sex | 0.80 | 0.30 | 0.32 | 2.63 | .01^**^ |
| Age | -0.01 | 0.01 | -0.04 | -0.57 | .57 |
| Religiosity | -0.24 | 0.03 | -0.48 | -7.54 | <.001^***^ |
| Intelligence | -0.07 | 0.06 | -0.07 | -1.10 | .27 |

*Notes:* Final Model: *F* (4, 195) = 17.67, *p* < .001, adjusted *R^2^* = .25. Coding: 0 = Male, 1 = Female. **p* < .05, ***p* < .01, ***p < .001

**Table S9**. Regression model that predicted the SSB scores of Mahayana Buddhists (N=200).

| Regressor | *B* | *SE B* | *β* | *t* | *P* |
| --- | --- | --- | --- | --- | --- |
| Intercept | -3.28 | 1.59 | .02 | -0.22 | .83 |
| Sex | -0.09 | .30 | -.04 | -0.31 | .76 |
| Age | -0.03 | .01 | -.14 | -2.27 | .02^*^ |
| Religiosity | 0.25 | .03 | .50 | 8.03 | <.001^***^ |
| Intelligence | -0.01 | .06 | -.01 | -0.14 | .89 |

*Notes:* Final Model: *F* (4, 195) = 19.85, *p* < .001, adjusted *R^2^* = .275. Coding: 0 = Male, 1 = Female. **p* < .05, ***p* < .01, ***p < .001

**Table S10**. Regression model that predicted the SSB scores of Christians (N=200).

**Replication Study**

To examine if our findings about the ToM ability are culture-specific to Taiwanese participants, we carried out a replication study with the U.S. population on Amazon Mechanical Turk (AMT). In total, 1750 AMT workers filled out our prescreening form and self-reported their religious affiliations. Among these workers, 8 workers (~1%) were Buddhists, 415 workers (24%) were Protestants, and 267 workers (~15%) were Catholics. Because Buddhists were too scarce on AMT and it was the negative correlation between Christians’ ToM scores and religiosity levels in our study that appeared contradictory to the previously observed positive relationship between mentalizing and religious belief in the literature, we hence followed up on only the Christian population, which included both Protestants and Catholics.

682 prescreened Christian workers were invited to participate in our online ToM study. Except being administrated in English, this AMT study was otherwise identical to the one reported in the main article. In the end, 186 workers (54 male, 132 female) completed the whole study without any missing values. Compared to the 200 Taiwanese Christian participants in the main study, there AMT workers were slightly lower in religiosity (M = 20.04, SD = 8.41) and intelligence (M = 15.52, SD = 5.19) and slightly higher in the ToM ability (M = 42.83, SD = 7.71).

| *Regressor* | *B* | *SE B* | *β* | *t* | *p* |
| --- | --- | --- | --- | --- | --- |
| Intercept | 31.36 | 3.12 | -.06 | -.50 | .62 |
| Sex | 0.66 | 1.13 | .09 | .58 | .56 |
| Age | 0.02 | 0.04 | .03 | .51 | .61 |
| Religiosity | -0.18 | 0.06 | -.20 | -3.07 | <.002^**^ |
| Intelligence | 0.46 | 0.11 | .31 | 4.36 | <.001^***^ |
| Perception | 0.59 | 0.18 | .24 | 3.26 | .001^**^ |

*Notes:* Final Model: *F* (5, 180) = 11.88 , *p* < .001, adjusted *R^2^* = .23. Coding: 0=Male, 1=Female. **p* < .05, ***p* < .01, ***p < .001

**Table S11.** Regression model that predicted the ToM scores of American Christians (N=186).
